# Microstructural Insights into Solid Dispersions: A Combined Small-Angle Neutron Scattering and Molecular Dynamics Approach

**DOI:** 10.1021/acs.molpharmaceut.5c01315

**Published:** 2026-02-07

**Authors:** Haoshi Gao, Yunsen Zhang, Hanqiu Jiang, Chunyong He, Yubin Ke, Haifeng Li, Defang Ouyang

**Affiliations:** † School of Pharmacy, 71237 Guangdong Pharmaceutical University, Guangzhou 510006, China; ‡ Institute of Applied Physics and Materials Engineering, University of Macau, Macau 999078, China; § State Key Laboratory of Quality Research in Chinese Medicine, Institute of Chinese Medical Sciences, 59193 University of Macau, Macau 999078, China; ∥ Faculty of Health Sciences, University of Macau, Macau 999078, China; ⊥ 71035 Institute of High Energy Physics, Chinese Academy of Sciences (CAS), Beijing 100049, China; # Spallation Neutron Source Science Center, Dongguan 523803, China

**Keywords:** small-angle neutron scattering, coarse-grained molecular dynamics simulations, solid dispersion, piroxicam, PEG

## Abstract

Solid dispersion is a widely adopted formulation strategy to enhance the solubility of water-insoluble drugs. However, the molecular-level structural determinants of stability and dissolution behavior remain poorly understood. This study integrates Small-Angle Neutron Scattering (SANS) technology with coarse-grained molecular dynamics (CGMD) simulations to investigate the effects of preparation methods (melting vs solvent evaporation) and drug loadings (10%, 15%, 25%) on the microstructure and crystallinity of PXM–PEG solid dispersions. Deuterated PEG (d-PEG) is used in the SANS to enhance the scattering intensity in samples. The findings revealed that the lamellar thickness decreased significantly from 173.01 Å (pure d-PEG) to 44.12 Å (25% drug loading, melting method), while the *d*-spacing reduced from 71.13 to 36.65 Å, indicating a substantial disruption of the crystalline structure. Conversely, samples prepared by solvent evaporation maintained larger *d*-spacing (up to 93.27 Å at 10% drug loading) and more stable layer stacking (Nlayers ∼5.6), demonstrating higher structural order. The results indicate that the preparation method significantly influences the structural characteristics of the solid dispersions. The melting method yielded a higher amorphous content at low drug loadings, which is expected to improve drug solubility and bioavailability. In contrast, the solvent evaporation method tended to produce solid dispersions with higher crystallinity and uniform structures at higher drug loadings. SANS results indicated that samples prepared by the melting method exhibited higher disorder in the high-*q* region, while those prepared by the solvent evaporation method showed greater crystallinity. The CGMD simulations further elucidated the dynamic aggregation and structural formation of the drug and polymer molecules during the preparation process. In the melting simulations, drug and polymer molecules gradually aggregated into dense clusters, while in the solvent evaporation simulations, the aggregates grew larger and more asymmetrical as the solvent evaporated, ultimately forming ordered structures. The combined results from SANS and molecular dynamics simulations indicated the “sandwich-like” structure of PXM–PEG solid dispersions. The outcomes of this innovative approach have the potential to advance the development of solid dispersion formulations, enhance research and development efficiency, and pave the way for the industrial production of solid dispersions.

## Graphical abstract

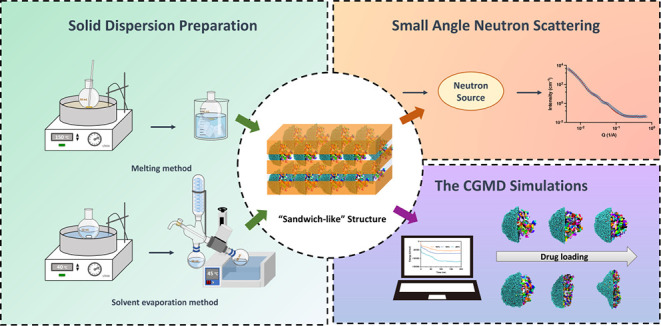

## 1 Introduction

Solid dispersions are a well-established pharmaceutical technology for improving the solubility and bioavailability of poorly soluble drugs.^1^ Various techniques such as melting, solvent evaporation, spray drying, coprecipitation, and hot melting extrusion can be employed to achieve this dispersion. The foundation of modern solid dispersion technology was established in 1961 when Sekiguchi and Obi published their groundbreaking work, introducing a novel approach to overcome solubility limitations of poorly soluble compounds through molecular dispersion in hydrophilic carriers.^2^ As a well-established solubilization technology with over six decades of development, solid dispersions are widely used in the pharmaceutical industry to increase the oral bioavailability of drugs *in vivo* by reducing the particle size of active pharmaceutical ingredients (APIs) and improving wettability and porosity to enhance the solubility.^3^

Despite the long history of using solid dispersions for drug delivery, the mechanisms underlying their formation remain poorly understood.^4^ Three structural hypotheses persist (see Figure 1): (1) amorphous drug molecularly dispersed in polymers, (2) crystalline drug embedded in a polymer matrix, and (3) amorphous drug domains within a polymer carrier.^1^

**Figure 1:**
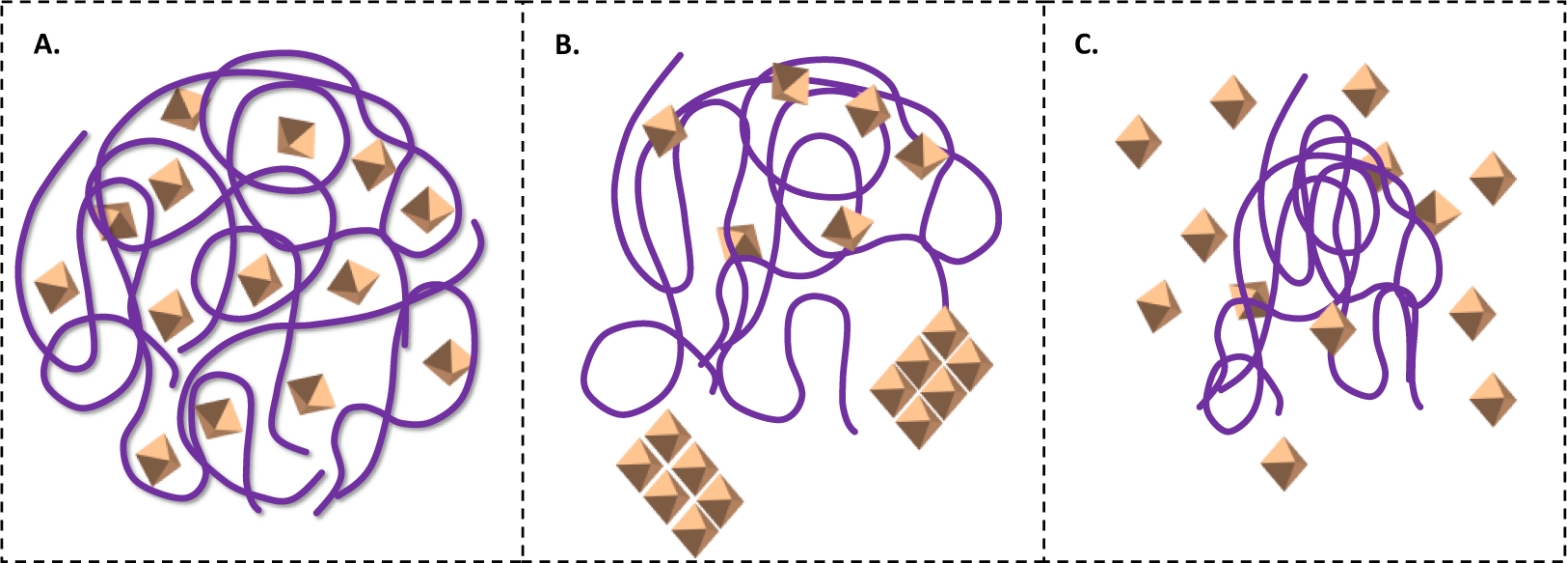
The diagram presents three potential structural configurations of a drug/polymer solid dispersion, where the octahedron shapes denote drug molecules and the wavy lines represent polymer chains: A. amorphous drug molecularly dispersed in polymers; B. crystalline drug embedded in a polymer matrix; C. amorphous drug domains within a polymer carrier.

Traditional characterization techniques-polarized light microscopy (PLM), X-ray diffraction (XRD) and differential scanning calorimetry (DSC)-focus on macroscopic crystallinity but lack nanoscale resolution.^5^ PLM can observe birefringence in crystallized samples, allowing for comparisons between the crystallization of drugs in solid dispersions and APIs.^6^ For instance, Onoue et al. found a reduction in the crystallinity of tranilast after forming a solid dispersion with poly­[MPC-*co*-BMA], which was accompanied by a decrease in particle size as observed by PLM.^7^ XRD provides valuable information about the crystallographic structure of samples and is widely used to analyze the transition of APIs in solid dispersions from crystalline to amorphous forms. For example, Khatri et al. demonstrated that the crystalline peaks of pyrimethanil diminish as the carrier amount increases, illustrating drug-carrier interactions.^8^ DSC, the most widely accepted thermal analysis technique, is often used to explore the structure of solid dispersions.^9^ de Souza et al. showed that hydrochlorothiazide transitions into an amorphous state during solid dispersion formation, as indicated by the disappearance of its melting point in DSC analysis.^10^ These examples highlight the utility of PLM, XRD, and DSC in studying the crystalline-to-amorphous transition in solid dispersions, although these primarily focus on macroscopic structures. However, these traditional techniques primarily analyze the macroscopic structure of solid dispersions and face challenges in characterizing the microstructure and understanding the complex mechanisms of drug-polymer interactions.

Small-angle neutron scattering (SANS) overcomes these limitations by probing nanoscale features (1–100 nm), including voids, dislocations, and phase separation.^11^ SANS is highly penetrating and allows nondestructive characterization to obtain detailed information about their microstructure.^12^ Developed in the 1960s as a complement to small-angle X-ray scattering (SAXS), SANS shares similar basic principles but has increasingly been used in the microstructural characterization of various materials.^13^ A key strength of SANS is its ability to modulate the scattering contrast, most notably through isotopic substitution. This is exemplified in biomedical studies, where the H_2_O/D_2_O ratio in the solvent is varied to label and resolve the distribution of protein and lipid within membranes.^14^ This powerful principle of contrast variation can be strategically extended to the study of solid dispersions. For semicrystalline polymer-based solid dispersions, such as the drug-PEG system studied here, a major analytical challenge is the intrinsically low scattering contrast between the drug and polymer carriers. We address this by employing fully deuterated PEG (d-PEG) as the matrix, which dramatically enhances neutron contrast between the crystalline and amorphous regions of d-PEG, and between the deuterated matrix and the hydrogenated drug.^15^

Additionally, molecular dynamics (MD) simulation provides a powerful *in silico* approach to exploring the microstructure of solid dispersions. Previous studies have focused on the mechanisms of formation, dissolution behavior, and physical stability of these dispersions.^16^ For example, our group first investigated the molecular structure of solid dispersions using simulated annealing, revealing more accurate molecular images than previous theories, with drug molecules either sticking to polymer coils or dispersing irregularly in low molecular weight carriers.^17^ Lately, we also used all-atom MD simulations to demonstrate how drug molecules gradually combine tightly with excipients, revealing the formation mechanism of solid dispersions at the atomic level.^18^ Moreover, we explored the dissolution mechanism of solid dispersions, providing a molecular dynamics landscape of drug–carrier interactions.^19^ MD simulations can also mimic accelerated conditions, such as high temperature and humidity, to investigate how polymers inhibit the recrystallization of APIs.^20^ While previous studies used all-atom MD simulations, the limitations of the all-atom model hinder the construction of large-scale solid dispersion models. Furthermore, advances in computational speed have made it clear that past results cannot accurately calculate larger systems due to these limitations. Despite these advancements, several limitations remain in the current MD simulation studies of solid dispersions. For instance, the scale of the initial structure in all-atom simulations is often too small to represent the entire solid dispersion, limiting the depiction of microstructures at the molecular level. Additionally, while all-atom simulations are well-suited for nanosecond time frames, the large number of atoms involved restricts their application to longer time scales.^21^ To address these challenges, coarse-grained molecular dynamics (CGMD) simulations offer a simplified model by grouping atoms or molecules into larger units, reducing computational complexity and allowing for the exploration of larger time and length scales.^22^ This approach is particularly suitable for studying the macroscopic behavior of solid dispersion systems, which involve complex, high molecular weight polymers.^23^

In this study, we aimed to utilize a multiscale approach by integrating SANS and CGMD simulations to investigate the structure of solid dispersion systems, as shown in Figure 2. Piroxicam (PXM) is used as the model drug, and d-PEG is used as the model polymer. The research begins with the characterization of the physical microstructure of solid dispersions and the molecular interactions between model drugs PXM and PEG. SANS will be utilized to elucidate the macroscopic microstructure of polymer solid dispersions, while CGMD simulations will delve into the intricate interactions between drugs and excipients at the molecular level. This work is expected to advance the development of solid dispersion formulations and ultimately hold considerable research significance and application value in promoting the industrialized production of solid dispersions.

**Figure 2:**
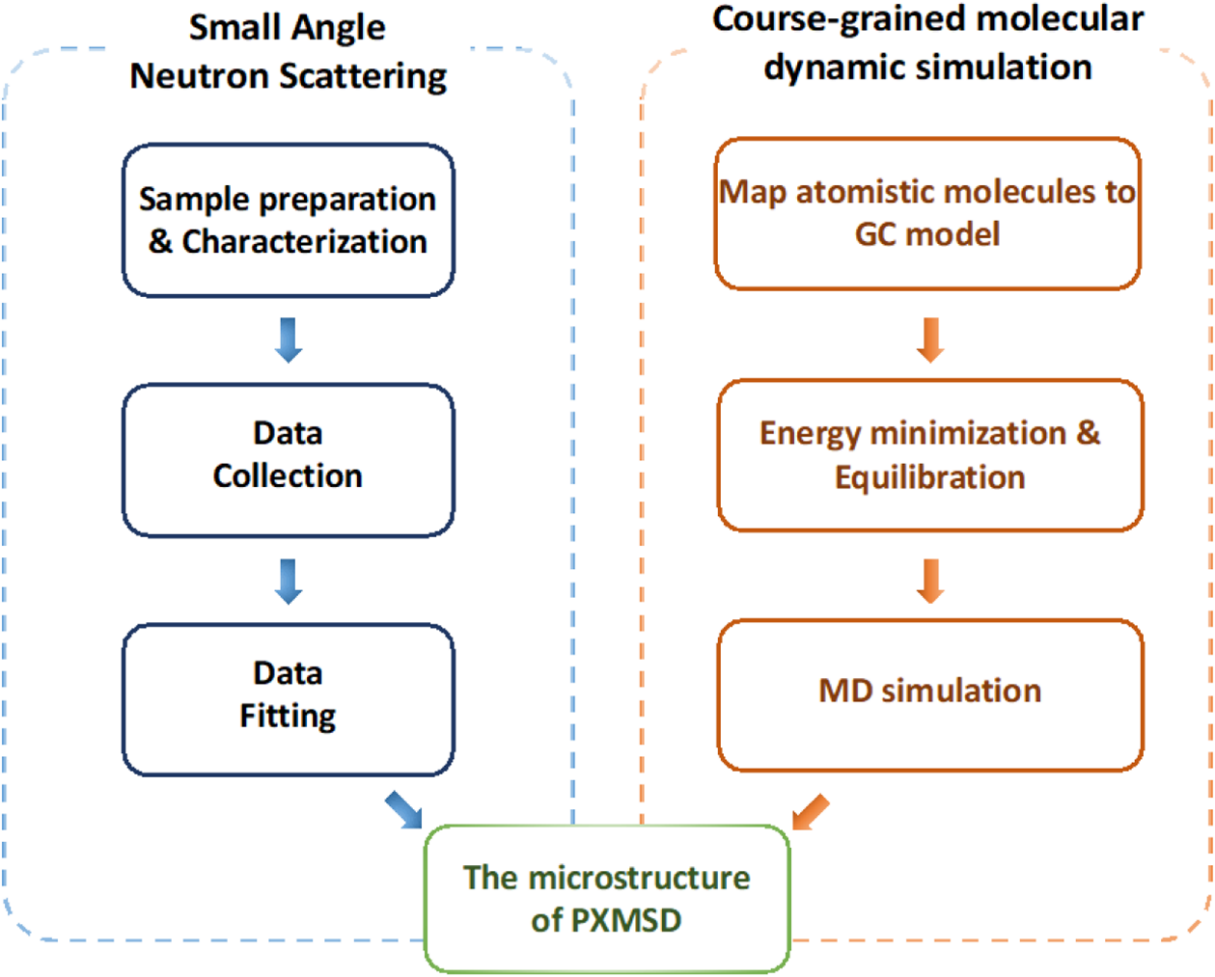
The flowchart of multiscale integrated SANS and CGMD investigates the microstructure of PXMSD.

## 2 Materials and Methods

### 2.1 Materials

PXM was purchased from Aladdin Chemical Co., Ltd. (Shanghai, China). Deuterated poly­(ethylene glycol) 6000 (dPEG) was obtained from Polymer Source, Inc. (Dorval, Canada).

### 2.2 Sample Preparation

The solid dispersion samples were prepared by melting and solvent evaporation methods. PXM and dPEG were weighted accurately at different drug loadings (10%, 15%, 25%) in both methods. In the melting method, PXM and dPEG, weighed in different mass ratios, are mixed in a preheated oil bath at 170 °C, with continuous stirring until a homogeneous molten mixture is formed. The mixture is then immediately quenched in an ice–water bath to rapidly cool and form an amorphous solid dispersion. After cooling, the solid mass is ground into a fine powder and dried in a vacuum oven at a low temperature to remove any residual moisture. Finally, the dried solid dispersion is stored in a desiccator or moisture-proof container for further use. The solvent evaporation method was similar to the study.^24^ Briefly, drugs and carriers were dissolved in warm ethanol, and the ethanol was removed by a rotavapor under 45 °C and vacuum. The residue was moved to a vacuum oven at room temperature overnight. Subsequently, the dispersion was smashed and passed through a 100-mesh sieve, and then stored in a dryer at room temperature.

### 2.3 Characterization

The powder X-ray diffraction (PXRD) patterns of the samples were analyzed using a powder X-ray diffractometer (Rigaku SmartLab, Japan) equipped with Cu Kα radiation (operating at 40 kV and 200 mA). The diffraction angle (2θ) was scanned from 10° to 60°, with a step size of 0.02°. SEM analysis of the PXM, piroxicam solid dispersion (PXMSD), and physical mixture was conducted using a ZEISS Sigma scanning electron microscope (ZEISS, UK). Each sample was securely mounted on stubs using conductive double-sided carbon tape and then coated with a thin layer of gold/palladium using a sputter coater for 90 s at 9 mA. The surface morphology of the samples was examined at an acceleration voltage of 5 kV.

### 2.4 Small-Angle Neutron Scattering

The SANS experiments were completed by a Small-Angle Neutron Scattering Instrument at the China Spallation Neutron Source (CSNS). All of the powder samples were loaded into 2 mm path length quartz cuvettes. For each sample measurement, the incident neutron wavelength (λ) was set from 1 to 9.8 Å, and the distance between samples and detector was 4 m with a 6 mm sample aperture. The *q* range of SANS measurement was 0.005 to 0.7 Å^–1^. Apart from the sample measurements, scattering contributions from the empty beam and empty cuvette were also collected and subtracted from the sample data. Additional corrections including sample thickness, transmission, detector efficiency, etc., were applied during the data reduction process, and the final sample scattering profiles were set to absolute scale based on a secondary standard sample, BatesPoly, provided by the beamline. The SANS data were analyzed using SasView 5 .0.5. All the data were fitted based on lamellar_stack_paracrystal, which was defined by M. Bergström.^25^ The polydispersity of the thickness was accounted for in the fitting routine and was represented by a Gaussian distribution. The eq 1 that defined this model, is as followed:

1 $$I\left(q\right)=2\pi \Delta \rho^{2}\Gamma_{m}\frac{P_{\mathrm{bil}}\left(q\right)}{q^{2}}Z_{N}\left(q\right)$$

In this model, the eq 2 form factor *P*_bil_(*q*) of the bilayer is approximated as a cross-section of an infinite, planar bilayer with thickness *t*.

2 $$P_{\mathrm{bil}}\left(q\right)={\left(\frac{\sin\left(qt/2\right)}{qt/2}\right)}^{2}$$

### 2.5 Computational Methods

#### 2.5.1 Coarse-Graining of Molecules

The coarse-graining procedure for the PXM and PEG6000 molecules was performed using the Martini 2.2 force field^26^ and computed using GROMACS2020. The PXM and PEG6000 were mapped onto a reduced representation where groups of atoms were represented by single coarse-grained. The PXM molecule was mapped according to its chemical structure, grouping nonpolar and polar regions into appropriate Martini bead types (e.g., SC4, SP1, and SNda). The PEG6000 was similarly coarse-grained, with repeated units represented by CG beads. The mapping followed the standard protocol provided by the Martini force field guidelines (see mapping rule as Figure S1 in Support Information). The parameter of ethanol was downloaded from Martini Force Field Initiative (https://cgmartini.nl/docs/downloads/force-field-parameters/martini2/solvents.html).

#### 2.5.2 Simulation System Setup

The PXM and PEG6000 were mixed in mass ratios of drug loading: 10%, 15%, and 25%. These mixtures were then solvated in a box containing coarse-grained ethanol beads. The dimensions of the simulation box were chosen to ensure sufficient space for the solutes and solvent, typically starting with a cubic box of 40 nm on each side. Bonded interactions, including bonds, angles, and dihedrals, were defined according to the Martini 2.2 force field. Nonbonded interactions were handled using Lennard-Jones potentials with standard Martini parameters. The cutoff for nonbonded interactions was set to 1.1 nm.

#### 2.5.3 Simulation Ensemble and Conditions

We first performed equilibration simulation of PXM monomer and PEG6000 monomer under canonical ensemble (NVT) to obtain the initial structures. The temperature was controlled using a velocity-rescaling thermostat set.^27^ The equilibrium simulation of an ethanol solvent box was performed under constant temperature and constant pressure conditions (NPT). The pressure was controlled using a Berendsen barostat set at 1 bar.^28^

#### 2.5.4 Melting Method

A simulation box containing the PXM molecules and PEG6000 in the specified mass ratios was constructed. The system was initialized at a temperature of 170 °C to mimic the heating conditions. Energy minimization was performed to remove any bad contacts in the initial configuration. The system was equilibrated at 170 °C under an NPT ensemble until the PXM was completely dissolved in the PEG6000 matrix. The temperature of the system was rapidly decreased to 0 °C to simulate the ice–water cooling process. The simulation continued until the mixture solidified completely. The temperature was maintained at 45 °C, and the drying process was simulated.

#### 2.5.5 Solvent Evaporation Method

A simulation box containing the PXM molecules and PEG6000 chains in the specified mass ratios was constructed and solvated by an equilibrated ethanol box. Ethanol molecules were added to the simulation box. Energy minimization was performed to remove any bad contacts in the initial configuration. The system was equilibrated at 40 °C under constant temperature and pressure (NPT ensemble) to ensure that the PXM and PEG6000 were well-mixed and dissolved in ethanol. Then, we removed all the ethanol molecules and performed simulations with the NVT ensemble. The temperature was maintained at 45 °C, and the drying process was simulated.

#### 2.5.6 Analysis

The final structures were analyzed to ensure a uniform dispersion of the PXM within the PEG6000 matrix. The interaction contacts between PXM and PEG6000 were analyzed to determine the extent of mixing and compatibility in the solid dispersions. The number of contacts between PXM and PEG6000 beads was calculated by using a distance cutoff of 0.6 nm. A contact was defined as any pair of beads within this distance. The number of contacts was compared across the different drug loading 10%, 15%, and 25% to assess how varying the PEG6000 concentration affected the mixing behavior. The radius of gyration (Rg) of the PXM and PEG6000 molecules was calculated to evaluate their spatial distribution and compactness within the PEG6000 matrix. The radius of gyration was calculated for each PXM molecule over the course of the simulation. Rg provides a measure of the overall size and shape of the molecule. The Rg was compared across different PEG6000 concentrations to assess the effect of PEG6000 on the PXM conformation.

## 3 Results

### 3.1 Characterization

#### 3.1.1 Scanning Electron Microscope (SEM)

Figure 3 presents the SEM images of pure PXM, PEG, physical mixtures, and solid dispersion samples, demonstrating significant variations in particle morphology depending on drug loading levels and preparation methodologies. For the melting method, the SEM image of PXMSD with 10% drug loading exhibited regular particle morphology, with smooth particle surfaces, uniform particle size, and minimal interparticle spacing. Increasing the drug loading to 15% resulted in moderately irregular particle shapes, expanded interparticle gaps, and slight size heterogeneity while maintaining relative surface smoothness. At 25% drug loading, particles displayed pronounced morphological irregularities characterized by rough surfaces with visible cracks, substantial size disparities, and markedly increased interparticle voids. In contrast, solvent evaporation-derived samples demonstrated progressive morphological deterioration across all drug loadings. Overall, the particles are irregular in shape, with rough surfaces and uneven sizes, and there are large gaps between them. At 10% drug loading, the particles have rough surfaces and large gaps; at 15% drug loading, the surface roughness increases, and the gaps become larger; at 25% drug loading, the particles are extremely rough, with significant size differences and even larger gaps.

**Figure 3:**
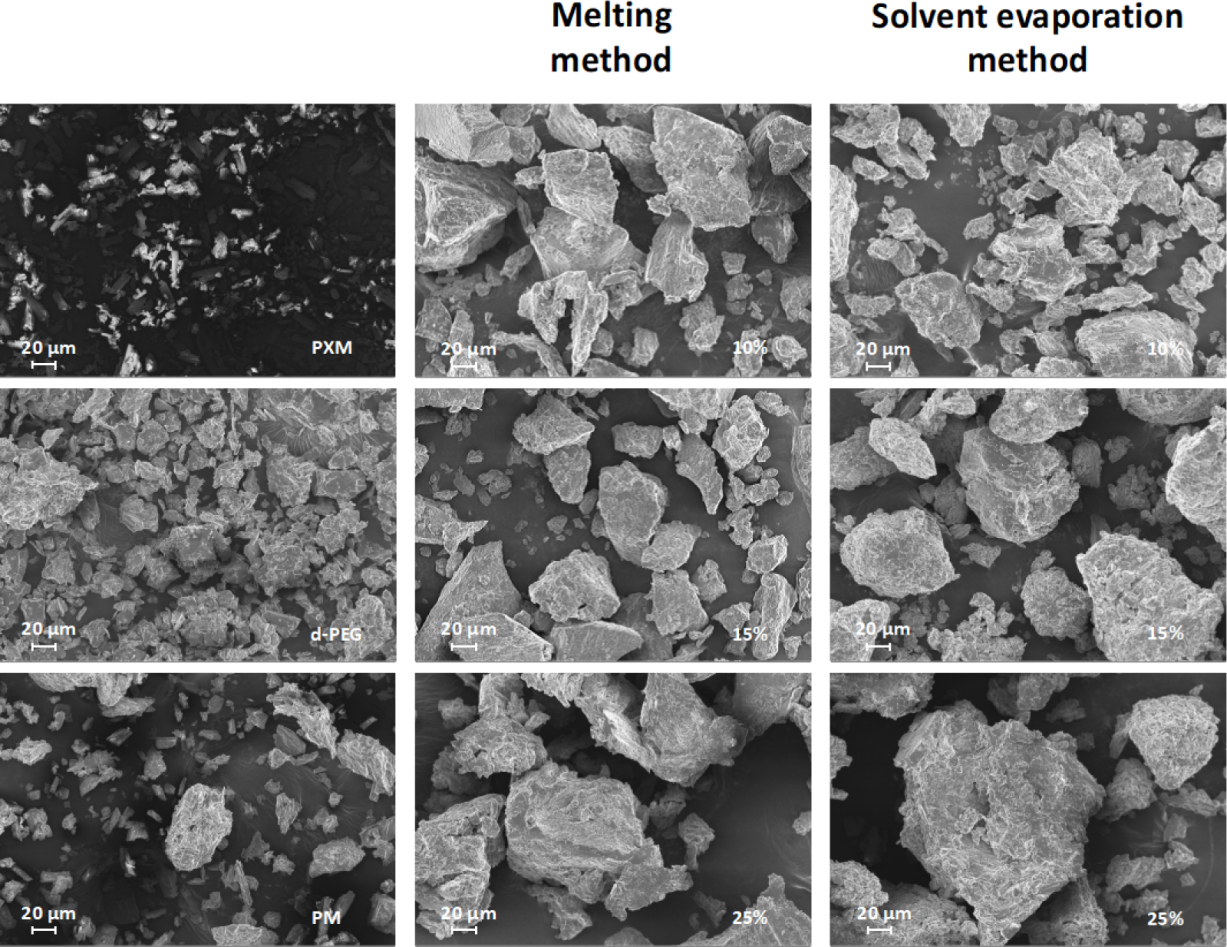
Comparison of morphological characteristics for PXMSD samples prepared by the melting method and solvent evaporation method.

#### 3.1.2 Powder X-ray Diffraction (PXRD)

Figure 4 displays XRD patterns revealing the distinct crystalline characteristics of the components. The characteristic diffraction peaks of pristine PXM and PEG correspond to their respective crystalline phases. The physical mixture pattern manifests as a simple superposition of individual component patterns, confirming the preservation of crystalline integrity in both drug and carrier. In the XRD patterns of samples with different drug loadings prepared by the melting method, a significant weakening or complete disappearance of PXM’s characteristic peaks was observed. This suggests that PXM had likely transitioned to an amorphous state, indicating the successful formation of a solid dispersion with PEG. Similarly, in the samples prepared by the solvent evaporation method, the observed weakening or absence of PXM’s characteristic peaks further corroborates the amorphization of the drug and the formation of a solid dispersion with PEG. The disappearance or weakening of these peaks indicates that the drug had transitioned to an amorphous state.

**Figure 4:**
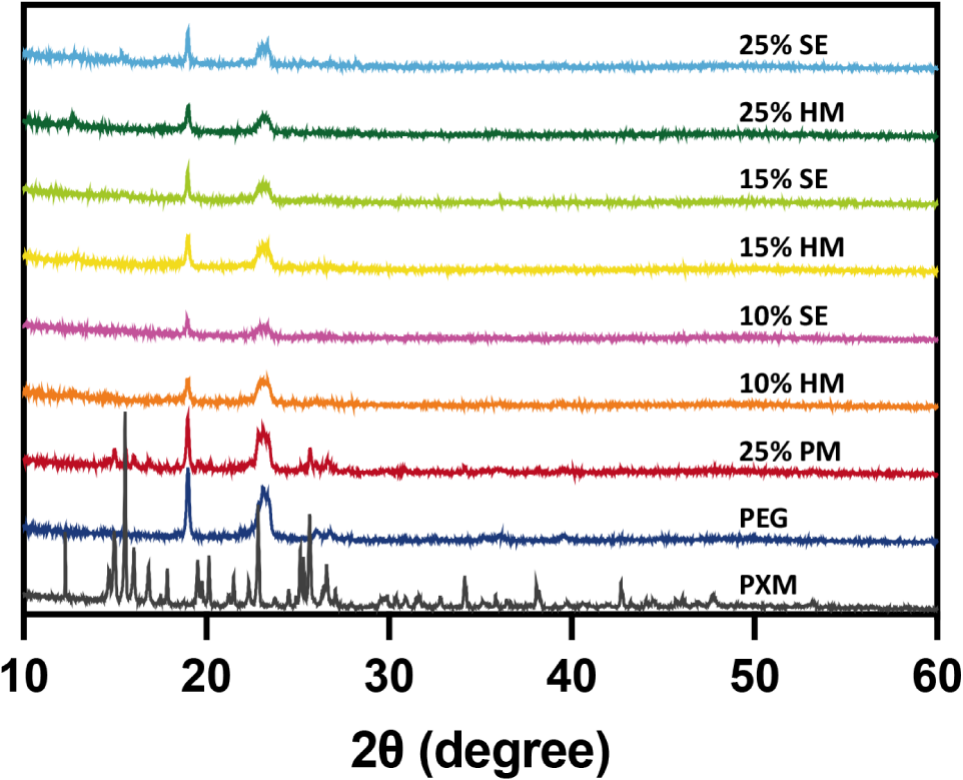
XRD patterns of pure PXM, PEG, physical mixture, and different drug-loading PXMSD prepared by the two methods.

### 3.2 Small-Angle Neutron Scattering

The microstructure of PEG and the API/PEG solid dispersion was investigated using SANS. Previous studies employing Small-Angle X-ray Scattering (SAXS) and Atomic Force Microscopy (AFM) have shown that PEG exhibits a lamellar spherulitic structure.^29^ After comprehensive evaluation of multiple fitting models, the lamellar_stack_paracrystal model was selected as the most suitable due to its fitting quality (evidenced by minimal χ^2^ values),^30^ effectively characterizing the periodic alternation of ordered crystalline lamellae and amorphous interlayers in semicrystalline polymeric systems. The model’s critical parameters (excluding fixed scattering length density values) include: thickness (crystalline lamella dimension perpendicular to the stacking direction), Nlayer (quantitative stacking multiplicity of crystalline domains), d_spacing (long-range stacking periodicity along the lamellar normal), and Sigma_d (polydispersity of the lamellar spacing).^25^ As shown in Figure 5A and B, the *q* ^*2*^ -dependent scattering profiles across all samples suggest a lamellar structural organization, as supported by the complete data set and fitting curves provided in Figure S2. This analytical framework provides essential insights into the hierarchical organization of solid dispersions, particularly elucidating crystalline–amorphous interface characteristics and stacking disorder phenomena.

**Figure 5:**
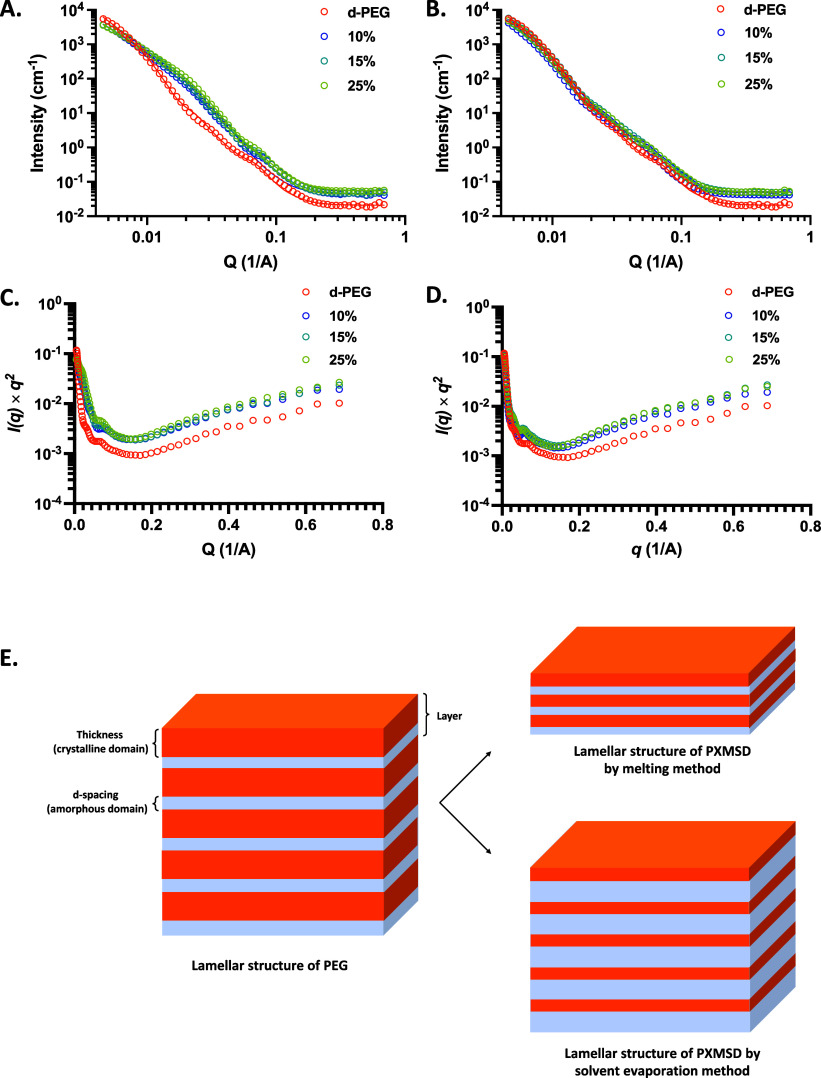
SANS profiles and fitting lines of A. melting method prepared PXMSD and B. solvent evaporation prepared PXMSD; Kratky plot of SANS for C. melting method prepared PXMSD and D. solvent evaporation prepared PXMSD; and E. schematic illustration (not to scale) of the lamellar structures for pure d-PEG and PXM solid dispersions prepared by melting and solvent evaporation methods.

Furthermore, SANS analysis of PXMSD samples prepared using two methods with three drug loadings (10%, 15%, and 25%) revealed method-dependent microstructure evolution. The low-*q* region was typically associated with large-scale structures;^31^ higher scattering intensity in this region suggests the presence of larger structural features such as aggregates or extensive amorphous domains. Conversely, a descending trend in the low-*q* region—observed across all samples—is indicative of nanoscale disordered structures.^32^ These findings provide complementary structural information across different length scales.

For the melting method, the SANS curve at 10% drug loading shows a rapid descent in the low-*q* region, indicating large-scale disordered structures such as amorphous regions. At the same time, a high scattering intensity in the high-*q* region suggests the presence of microstructures or small-scale aggregates. As drug loading increases to 15%, the curve maintains a similar shape but with higher overall intensity, indicating that the amorphous and disordered regions have become more pronounced. At 25% drug loading, the curve descends more slowly in the low-*q* region, suggesting a reduction in large-scale amorphous regions and improved structural uniformity; simultaneously, the slightly decreased intensity in the high-*q* region indicates a slight reduction in the disorder of small-scale structures.

To further probe the microstructure of the solid dispersion, Kratky analysis was performed. The resulting Kratky plot of d-PEG exhibits a sharp decrease in the low-*q* region (0–0.02 Å^– 1^), followed by a pronounced decline in *q* ^*2*^ *I­(q)* values to a minimal plateau (≈0.003–0.0018) between 0.02 and 0.07 Å^– 1^. Above *q* > 0.1 Å^– 1^, *q* ^*2*^ *I­(q)* increases gradually from approximately 0.001 to 0.005–0.01, reaching about 0.01 as *q* approaches 0.7 Å^– 1^. In contrast, the PXMSD prepared by the melting method displayed a notably different profile in the Kratky plot. Pure d-PEG exhibits the highest value (≈0.117). After drug loading, values decrease significantly (10–25% drug loading: ≈0.077–0.078), with minimal variation between different drug loading amounts. Pure d-PEG exhibits the steepest decay slope; the slope flattens after drug loading and becomes increasingly gradual with increasing drug loading amounts. Drug incorporation immediately and significantly disrupts PEG’s original large-scale ordered structure.^33^ Suppression of the low-*q* signal approaches saturation at 10% drug loading with no further significant changes observed at higher drug loading amounts. While a similar decreasing trend was observed in the low-*q* region (0–0.02 Å^– 1^), the initial *q* ^2^ *I­(q)* values were lower, and the subsequent plateau between 0.02 and 0.07 Å^– 1^ was shallower and higher (≈0.003). In the high-*q* region (*q* > 0.1 Å^‑1^), *q* ^2^ *I­(q)* exhibits a pronounced upward trend of the scattering intensity with drug loading. The end point values rise progressively from 0.010 for pure d-PEG to 0.019 (10% drug loading), 0.023 (15% drug loading), and 0.027 (25% drug loading). This indicates that high-contrast interfaces on the small scale (1–10 nm), such as drug-carrier interfaces or drug-rich nanoclusters, continuously increase or intensify with higher drug loading, reflecting a gradual enhancement of drug dispersion or phase-separation behavior at the nanoscale.^34^ The corresponding fitting parameters of lamellar_stack_paracrystal listed in Table 1 revealed a progressive microstructural change with increasing drug loading evolution. The background scattering intensity of samples ranged from 0.019 to 0.051 cm^– 1^, indicating fewer noncharacteristic scattering signals, which enhances the ability to analyze the microstructure of solid dispersions. The pure d-PEG exhibited high thickness, more layers, consistent layer spacing, and a low standard deviation. These features indicate a well-defined crystalline region with a uniform lamellar structure. Compared to pure PEG (d-PEG), the thickness of the samples prepared using the melting method decreased from 173.01 to 44.12 Å as the drug loading increased, while the number of layers reduced from 5.61 to approximately 3. Additionally, the *d*-spacing value was halved. To visualize the evolution of the microstructure, Figure 5E schematically illustrates that the solid dispersions prepared by the melting method contained fewer lamellar layers and exhibit an expanding amorphous fraction with increasing drug loading. Notably, the parameter sigma_d, which described the polydispersity of lamellar spacing, reaches values of 1.76 and 1.64 for the 10% and 15% drug loading PXMSD samples, significantly higher than that of pure d-PEG. At 25% drug loading, the sigma_d decreases to 0.42, which is slightly lower than that of pure d-PEG. This indicates the formation of a highly uniform lamellar structure within the solid dispersion.

**Table 1:** SANS Parameter of PXMSD Fitting by the lamellar_stack_paracrystal Model

Samples	Background (cm^–1^)	SLD (10^–6^/Å^2^, fixed)	Thickness (Å)	Nlayers	d_spacing	Sigma_d
d-PEG	0.019	6.45	173.01 ± 0.46	5.61 ± 0.004	71.13 ± 0.05	0.54 ± 0.0015
d-SD 10%-HM	0.046	6.13	99.95 ± 0.13	3.03 ± 0.002	41.5 ± 0.97	1.76 ± 0.064
d-SD 15%-HM	0.040	5.98	100.64 ± 0.28	3.24 ± 0.002	31 ± 0.84	1.64 ± 0.072
d-SD 25%-HM	0.050	5.63	44.12 ± 0.054	3.3 ± 0.001	36.65 ± 0.01	0.42 ± 0.001
d-SD 10%-SE	0.050	6.13	170.51 ± 0.31	4.45 ± 0.004	93.27 ± 0.071	0.61 ± 0.001
d-SD 15%-SE	0.050	5.98	97.26 ± 0.15	5.66 ± 0.003	70.64 ± 0.021	0.50 ± 0.0007
d-SD 25%-SE	0.051	5.63	91.50 ± 0.14	5.87 ± 0.003	71.09 ± 0.02	0.50 ± 0.0007

The Kratky plots of the solid dispersions prepared by the solvent evaporation method exhibit a distinct “U-shaped” profile, characterized by a relatively high initial intensity in the low-*q* region (*q* < 0.02 Å^– 1^), a broadened and elevated plateau in the mid-Q range (0.02–0.1 Å^– 1^), and a significant rise in the high-*q* region (*q* > 0.1 Å^– 1^) that peaks at 15% drug loading before slightly decreasing at 25% drug loading. Unlike the melt method, the solvent-processed samples retain much of the large-scale order of the d-PEG matrix, as evidenced by only moderate reduction in low-*q* scattering upon drug incorporation. The pronounced elevation of the mid-*q* plateau indicates enhanced heterogeneity at intermediate length scales (∼10–30 nm), likely due to solvent-mediated phase separation. The nonmonotonic trend in the high-*q* rise—strongest at 15% drug loading, which suggests an optimal drug loading window for generating nanoscale interfaces, beyond which phase coarsening or altered aggregation may reduce the small-scale contrast. These features collectively reflect the kinetically controlled, solvent-driven microstructure evolution, which preserves long-range lamellar order while introducing tunable nanoscale heterogeneity. Combined with the lamellar model fitting results for the solvent-evaporated samples, the nonmonotonic variations observed in the Kratky plots are consistently explained by quantitative structural parameters. At 10% drug loading, the lamellar thickness remains essentially unchanged (170.5 Å), whereas the interlayer spacing expands markedly to 93.3 Å, suggesting that drug molecules are mainly intercalated into the amorphous regions without severely disrupting the crystalline lamellae. At 15% loading, the thickness decreases to 97.3 Å, while the stacking layers recover to 5.66 and the *d*-spacing returns to 70.6 Å. Coupled with a low sigma_d value, these features indicate the formation of a well-ordered, periodic thin-layer stack, resembling the structural regularity of pure d-PEG. These structural changes correspond directly to the recovery of low-*q* intensity (enhanced long-range order) and the pronounced high-*q* rise (maximum density of nanoscale interfaces) seen in the Kratky plot.^35^ When the drug loading increases to 25%, the structural parameters become nearly identical to those at 15% (thickness = 91.5 Å, layer number = 5.87, *d*-spacing = 71.1 Å), signifying that the system has reached a uniform and stable structural state. In summary, the solvent evaporation process is governed by kinetic, solvent-mediated pathways that facilitate an ordered structural reorganization at intermediate drug loadings.^36^ This reorganization reduces the individual layer thickness while restoring stacking periodicity, thereby introducing tunable nanoscale interfaces within a preserved long-range lamellar framework. As schematically illustrated in Figure 5E, the ordered lamellar architecture of the solvent-evaporated PXMSD confines the drug principally within the amorphous interlayers, enabling nanoscale phase separation while preserving the long-range stacking order of the carrier matrix.

### 3.3 Molecular Dynamic Simulation Results of the PXMSD

Figure 6 demonstrated the use of coarse-grained molecular dynamics simulation to observe the structural changes in solid dispersions with different drug loadings (10%, 15%, 25%) during the melting process from 0 ns to 300 ns. At 0 ns, drug and polymer molecules were evenly distributed. Over time, drug molecules and polymer clusters started to aggregate, forming small clusters. By 300 ns, drug molecules had further aggregated into larger, denser clusters with polymer molecules, resulting in relatively distinct drug-polymer spherulite structure. The density profiles indicate that the drug was primarily distributed within the PEG matrix, and as drug loading increased, the overall density of the solid dispersion system decreased.

**Figure 6:**
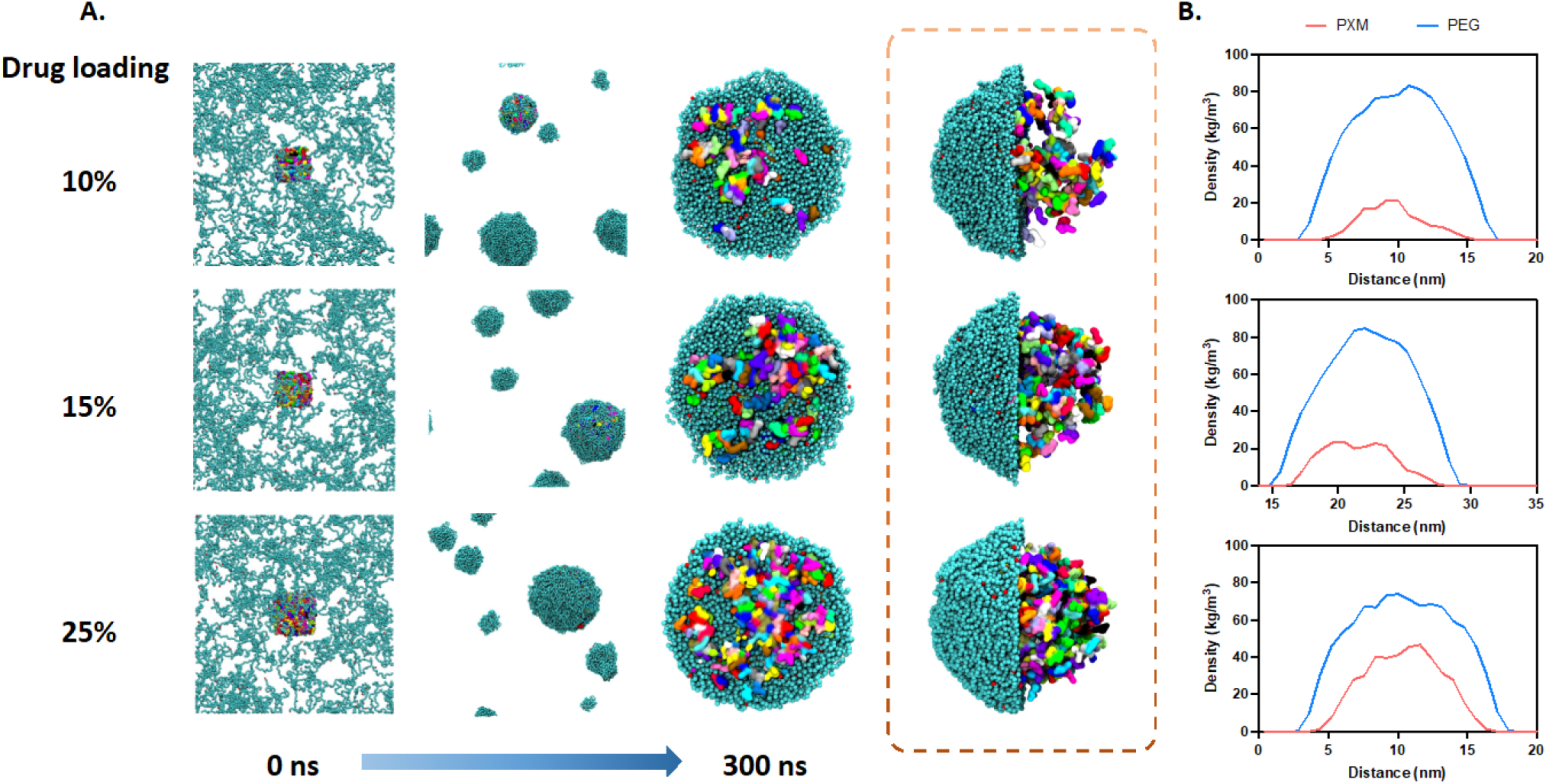
The MD process of PXMSD formation by the melting method. A. Snapshots taken from CGMD simulations of 10% to 25% drug-loading systems from 0 to 300 ns; B. the density profiles of 10% to 25% drug-loading systems.

Meanwhile, Figure 7 illustrates the structural changes in solid dispersions with different drug loadings (10%, 15%, and 25%) during the solvent evaporation process using coarse-grained molecular dynamics simulations. The simulation process was divided into two stages: the first stage involved reactions in the ethanol from 0 to 100 ns, and the second stage involved the removal of the ethanol molecules to simulate the solvent evaporation process from 100 to 200 ns. At the first stage, PXM molecules (colored) and PEG molecules (blue) were observed evenly dispersed in the ethanol medium. With ethanol molecules evaporated, drug molecules and PEG molecules aggregated into clusters of varying shapes and sizes depending on the drug loading. Higher drug loadings (25%) resulted in more extensive and irregular elliptical spherulite structures . The density profile also confirmed such structural features.

**Figure 7:**
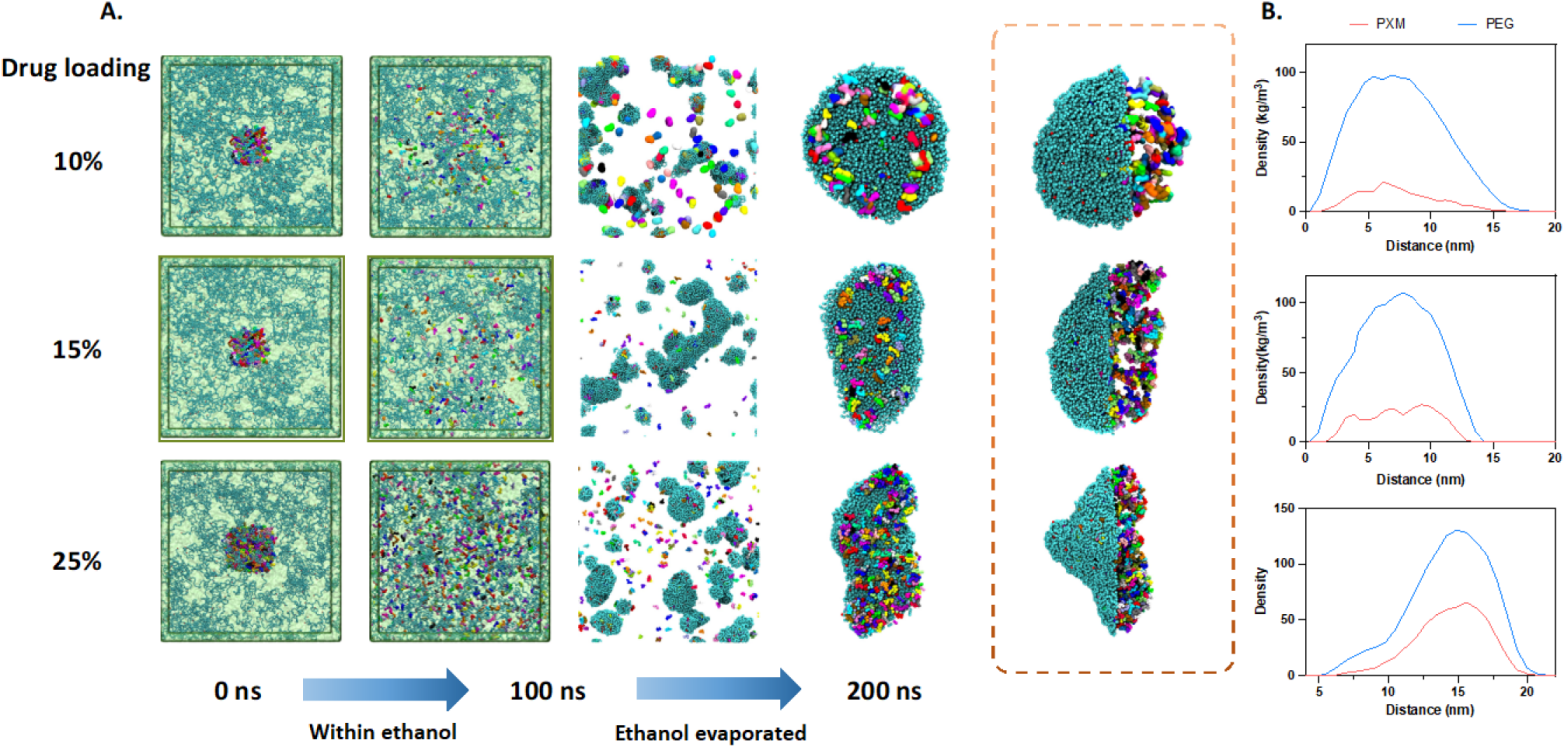
The MD process of PXMSD formation by solvent evaporation method. A. Snapshots taken from CGMD simulations of 10% to 25% drug-loading systems from 0 to 300 ns; B. the density profiles of 10% to 25% drug-loading systems.

Figure 8 and Table S1 present the energy variation plots and potential energy values, illustrating the interactions between drugs and polymers at varying concentrations, as well as the energy evolution under two preparation methods: the melting method and the solvent evaporation method, as analyzed by CGMD. Specifically, in the melting method, the drug–drug interaction energy initially increased and then stabilized, indicating that the drug molecules gradually reached a stable interaction state under thermal conditions. As the drug loading increased from 10% to 25%, the drug–drug interaction energy significantly decreased from −18167.3 to −83043.6 kJ/mol, indicating that the strength of the interactions between drug molecules increased with higher loading (see Table S1). At higher drug concentrations, drug molecules tended to aggregate, resulting in stronger interaction forces. In contrast, the interaction energy decreased rapidly in the solvent evaporation method, indicating that drug molecules formed strong interactions as the solvent evaporated. At higher drug loadings, the lower energy values suggested more compact molecular binding. Although the solvent evaporation method followed a similar trend to the melting method, the interaction energies were smaller. For instance, at 25% drug loading, the drug–drug interaction energy was −52262 kJ/mol, indicating weaker drug–drug interactions compared with the melting method, as shown in Table S1. This difference could be attributed to the solvent evaporation process, which promotes a more uniform dispersion of drug molecules, thereby reducing the likelihood of drug aggregation.

**Figure 8:**
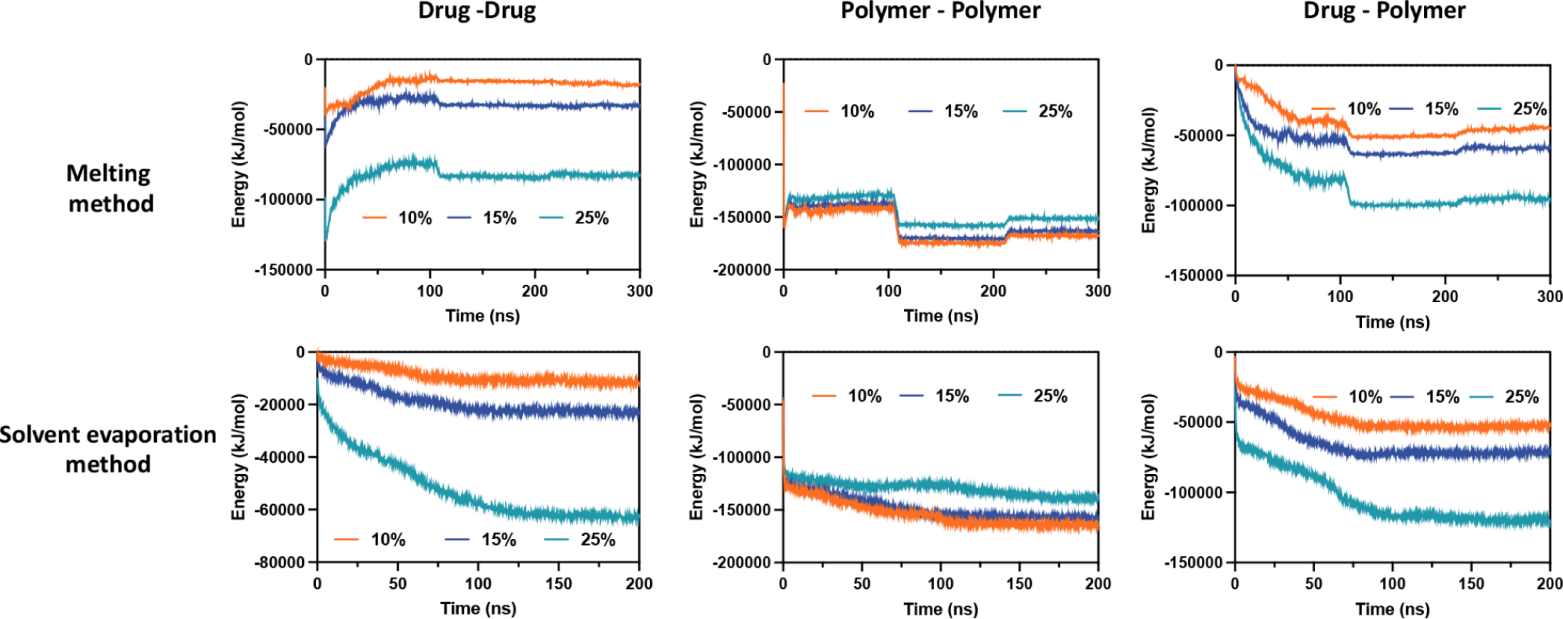
The potential energy profile of different drug-loading PXMSD prepared by two methods by CGMD.

For polymer–polymer interactions, in the melting method, the energy variation was relatively small, indicating gradual polymer interactions. As drug loading increased, the polymer–polymer interaction energy decreased from −161148 to −146265 kJ/mol, suggesting that the interactions between polymer chains weakened, possibly due to interference from drug molecules disrupting the close packing of the polymer chains. This result was in good agreement with the SANS result, in which the PXM changed the microstructure of PEG, forming a tighter structure with less layer. In the solvent evaporation method, the polymer–polymer interactions were weaker compared to those in the melt method. This was because the solvent evaporation process reduced the interactions between polymer chains, which, in turn, facilitated a more homogeneous drug dispersion. As the drug loading increased, the polymer–polymer interaction energy rose from −154,030 to −129,617 kJ/mol. The increased drug loading enhanced the interaction forces between PXM and PEG, contributing to the formation of a stable solid dispersion. Additionally, the increase in PXM led to structural changes in PEG. The SANS results showed that the solid dispersion formed by the solvent evaporation method had a similar number of lamellar layers to PEG, but with a decrease in crystalline regions and an increase in amorphous regions.

For drug-polymer interactions, in the melting method, the interaction energy significantly decreased, indicating that drug molecules progressively interacted with polymers during the heating process, with stronger interactions observed at higher drug loadings, particularly at 25%. In the solvent evaporation method, the energy declined rapidly, suggesting that drug molecules quickly bound with polymers as the solvent evaporated, with stronger interactions similarly observed at higher drug loadings. In the melting method, the drug-polymer interaction energy decreased from −42045.9 kJ/mol at 10% to −87186.7 kJ/mol at 25%, indicating that as drug loading increased, the interactions between the drug and polymer became stronger, especially at 25%, where these interactions were the most pronounced. This enhanced interaction could improve the stability of the drug and prevent recrystallization. A similar trend was observed in the solvent evaporation method, where the drug-polymer interaction energy decreased from −47328.3 kJ/mol at 10% to −104679 kJ/mol at 25%. Compared to the melting method, the drug-polymer interaction energy was lower in the solvent evaporation method, indicating tighter binding between the drug and polymer at higher loadings, which can enhance the drug’s solubility and physical stability.

Figure 9 presents the molecular simulation results of the interactions between PXM and PEG at different drug loadings (10%, 15%, and 25%) under two preparation methods. For the melting method, the number of interactions for the three drug loadings increased rapidly in the initial stage and stabilized at around 5 × 10^6^, 1.0 × 10^7^, and 1.3 × 10^7^, respectively. Concurrently, for the solvent evaporation method, the number of interactions for the three drug loadings increased rapidly and stabilized at around 10,000, 20,000, and 40,000 in the first stage (0 to 100 ns). As ethanol gradually evaporated, the number of interactions increased rapidly but with significant fluctuations, finally stabilizing at around 100, 200, and 400, respectively. These fluctuations were mainly due to the gradual evaporation of ethanol, leading to the rearrangement and aggregation of polymer chains and drug molecules, causing substantial dynamic changes. Higher drug loadings resulted in more interactions.

**Figure 9:**
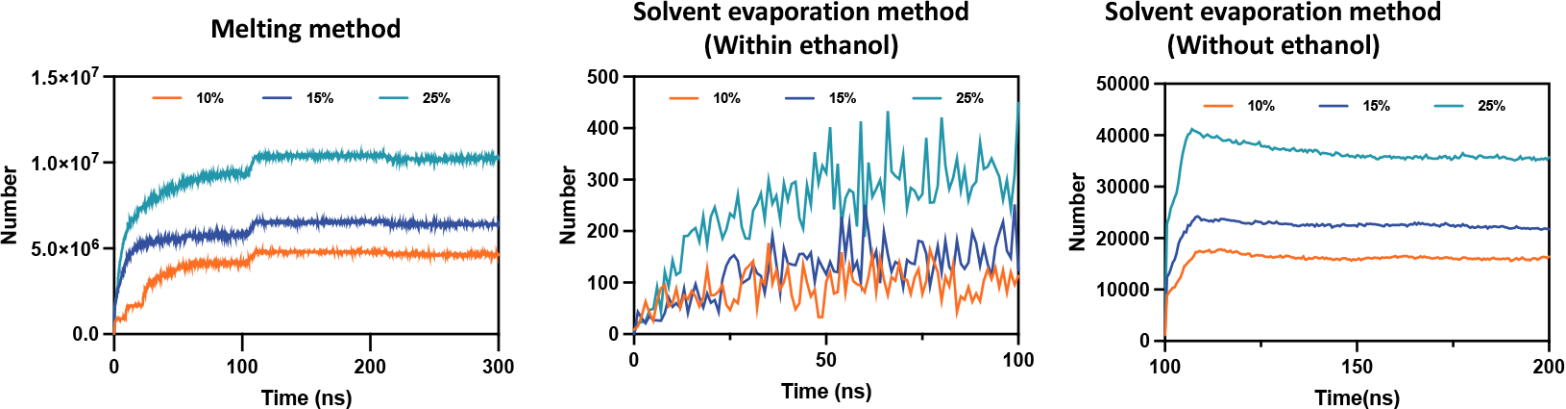
The count of interactions between drugs and polymers.

## 4 Discussion

The integration of Small-Angle Neutron Scattering (SANS) and coarse-grained molecular dynamics (CGMD) simulations provides a comprehensive, multiscale perspective on the microstructural evolution of PXM–PEG solid dispersions. SANS quantifies nanoscale structural parameters (lamellar thickness, *d*-spacing, Nlayers, and sigma_d), while CGMD simulations reveal the underlying molecular interactions and aggregation dynamics that govern these structural features.

From the macroscopic view provided by SANS data, as drug loading increases, there is a notable reduction in layer spacing, thickness, and number of layers, particularly in the samples prepared by the melting method. For higher drug loadings, the layer spacing and thickness significantly decrease, indicating that drug molecules are increasingly disrupting the polymer’s ordered lamellar structure, leading to a transition toward a more amorphous state. In contrast, in the solvent evaporation method, the samples retain more of their ordered structure, with relatively larger layer spacing and thickness, suggesting that this method helps preserve more of the polymer’s crystalline organization, even at higher drug loadings. These observations suggest that the polymer matrix forms a lamellar framework that can be disrupted or preserved depending on the preparation method and drug loading.

Complementing these macroscopic observations, CGMD simulations provide microscopic insights into the molecular mechanisms governing structural changes. In our past research, the structure of solid dispersion was proven to show that linear polymer chains form random coils under heat, with drug molecules adhering to their surface, while drug molecules disperse irregularly in amorphous low molecular weight carriers.^17^ However, in this study, with advancements in computational speed and molecular dynamics simulation technology, we used GCMD and longer PEG chains to build a larger system, resulting in findings that differ from those of our previous results.

The simulations show that the number of drug-polymer interactions increases significantly with a higher drug loading, especially in the melting method samples. This rise in interactions directly corresponds to the disruption of the polymer’s crystalline structure observed in the SANS data. In contrast, in the solvent evaporation method, both the number of interactions and energy changes are more stable, indicating that drug molecules are less disruptive to the polymer’s ordered structure, which is reflected in the larger layer spacing and greater thickness seen in the SANS data. This suggests that the solvent evaporation method promotes a more uniform distribution of spherical drug aggregates within the lamellar polymer framework, preserving the overall crystalline order.

A systematic correlation between SANS parameters and CGMD-derived metrics further validates this multiscale consistency. For instance, the significant reduction in lamellar thickness (from 173.01 to 44.12 Å) and decrease in the number of layers with increasing drug loading in the melting method, as quantified by SANS, are consistent with the CGMD results showing enhanced drug-polymer interactions (evidence by the decrease in drug-polymer interaction from −42,045.9 kJ·mol^–1^ at 10% loading to −87,186.7 kJ·mol^–1^ at 25% loading) and increased drug aggregation (reflected in the more negative drug–drug interaction energy). Conversely, the solvent-evaporation method preserved large d_spacing (up to 93.27Å) and higher Nlayers (≈ 5.6), which correlated with weaker drug–drug interactions and a more uniform dispersion of drug molecules in the MD simulations. Furthermore, the drug-polymer interaction energy was most negative for the 25% drug loading formulation, indicating the strongest intermolecular binding. This, coupled with its low Sigma_d value (approaching that of pure d-PEG), confirms it as the most structurally stable and tightly integrated formulation. While CGMD simulations successfully captured the general trend of potential energy changes across samples prepared by both methods, it is important to note that such simulations are inherently based on idealized models. A distinct experimental observation from SANS was the relatively high Sigma_d values (>1.64) for low-drug-loading (10–15%) samples prepared via the melting method. This result suggests a practical processing consideration: achieving a homogeneous molecular-level dispersion at low drug concentrations during the short melting and quenching cycle may be kinetically constrained, which could contribute to the observed nanoscale structural variation and higher polydispersity in the solid dispersion.

The multiscale structural insights obtained in this study allow for a critical reevaluation of the three classical hypotheses concerning drug-polymer solid dispersions. While Hypothesis B (crystalline drug embedded in a polymer matrix) is unequivocally refuted by the absence of distinct drug crystalline signatures in PXRD patterns, our findings provide nuanced support and substantial refinement for Hypotheses A and C. The data suggest that the system prepared by the melting method, particularly at low drug loading, approaches the state described in Hypothesis A (amorphous drug molecularly dispersed in polymers), as evidenced by strong drug-polymer interactions and high apparent dispersion from CGMD simulations. However, the significant disruption of the PEG lamellar order and the elevated interfacial polydispersity (Sigma_d) revealed by SANS indicate that this state is not a perfectly homogeneous molecular dispersion but rather a nanoscopically heterogeneous system. This observation leads to our proposed “sandwich” structural model, which refines Hypothesis A by specifying that the dispersed drug is predominantly localized within the amorphous interlayers of the semicrystalline matrix rather than being uniformly distributed throughout the entire polymer volume. Most conclusively, our work provides direct multiscale validation and spatial elaboration of Hypothesis C (amorphous drug domains within a polymer carrier). As evidenced by the evolution of SANS parameters such as *d*-spacing, layer number, and Sigma_d, these amorphous domains are systematically confined within the periodic amorphous galleries of the lamellar PEG architecture, demonstrating an ordered arrangement rather than random organization. This confined, periodic arrangement of drug-rich amorphous regions between crystalline PEG layers constitutes the essence of the “sandwich” structure. Furthermore, the methodology plays a decisive role in realizing these structural states: the solvent evaporation method proved to be the most effective pathway for achieving a well-ordered, periodic “sandwich” architecture.

In summary, the combination of SANS and CGMD simulations elucidates how preparation methods and drug loading influence the solid dispersion microstructure. The melting method, with stronger drug-polymer interactions, disrupts the lamellar order and fosters amorphous drug aggregation. The solvent evaporation method promotes a more uniform distribution of the drug within the preserved lamellar framework. The resulting “lamellar-spherical” composite structure has direct implications for performance: the lamellar scaffold contributes to physical stability, while the confined amorphous drug domains enhance solubility. This multiscale insight provides a rational basis for optimizing solid dispersion formulations.

## 5 Conclusion

The comprehensive analysis of SANS and molecular dynamics simulations has provided significant insights into the “sandwich-like” microstructure of solid dispersions. MD simulation results are complemented by SANS results, providing a comprehensive perspective on the microstructural changes in solid dispersions. CGMD simulations revealed the dynamic process of drug-polymer interactions and aggregation behavior under different preparation methods and drug loadings, while SANS offered a microscopic view of these structures. By integrating these results, more accurate guidance can be provided for optimizing the preparation of solid dispersions to enhance drug solubility, bioavailability, and stability. This combined approach was crucial for the development of effective, safe, and controlled drug formulations.

## Supplementary Material



## Notes

The mapping rule of Piroxicam and PEG6000 in Martini 2.2 force field is shown in Figure S1. SANS data with fitted curves are provided for: PXM solid dispersions prepared by the melting method; PXM solid dispersions prepared by the solvent evaporation method; and pure d-PEG, as shown in Figure S2. The potential energy of drug–drug, polymer–polymer, and drug–polymer interactions for different drug loadings under the melting and solvent evaporation methods is presented in Table S1 (PDF)
