# Supplementary material for: Microstructural Insights into Solid Dispersions: A Combined Small-Angle Neutron Scattering and Molecular Dynamics Approach
Source: Mol Pharm. 2026 Feb 7;23(4):2350–61. doi: 10.1021/acs.molpharmaceut.5c01315 (PMC13058892; doi:10.1021/acs.molpharmaceut.5c01315)
Supplement: Supplementary file 1 [file mp5c01315_si_001.pdf]

Supporting information for

Microstructural Insights into Solid Dispersions: A Combined Small Angle Neutron Scattering and Molecular Dynamics Approach

Haoshi Gao[a, c, 1], *Yunsen Zhang*[c, d, 1], *Hanqiu Jiang*[e, f], *Chunyong He*[e, f], *Yubin Ke*[e, f], *Haifeng Li*[b, *], *Defang Ouyang*[c, d, *]

[a] *School of Pharmacy, Guangdong Pharmaceutical University, Guangzhou 510006, China*

[b] *Institute of Applied Physics and Materials Engineering, University of Macau, Macau 999078, China*

[c] *State Key Laboratory of Quality Research in Chinese Medicine, Institute of Chinese Medical Sciences, University of Macau, Macau 999078, China*

[d] *Faculty of Health Sciences, University of Macau, Macau 999078, China*

[e] *institute of High Energy Physics, Chinese Academy of Sciences (CAS), Beijing 100049, China*

[f] *Spallation Neutron Source Science Center, Dongguan 523803, China*

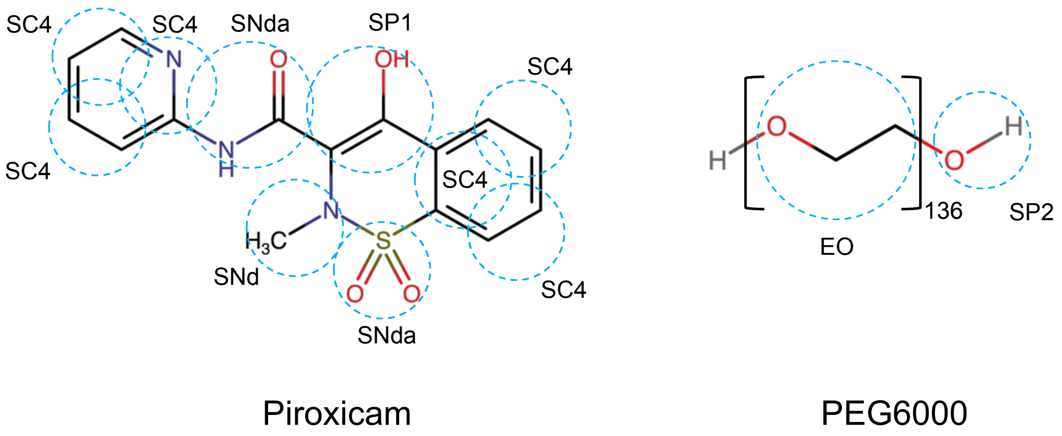

Figure S1. The mapping rule of Piroxicam and PEG6000 in Martini 2.2 force field.

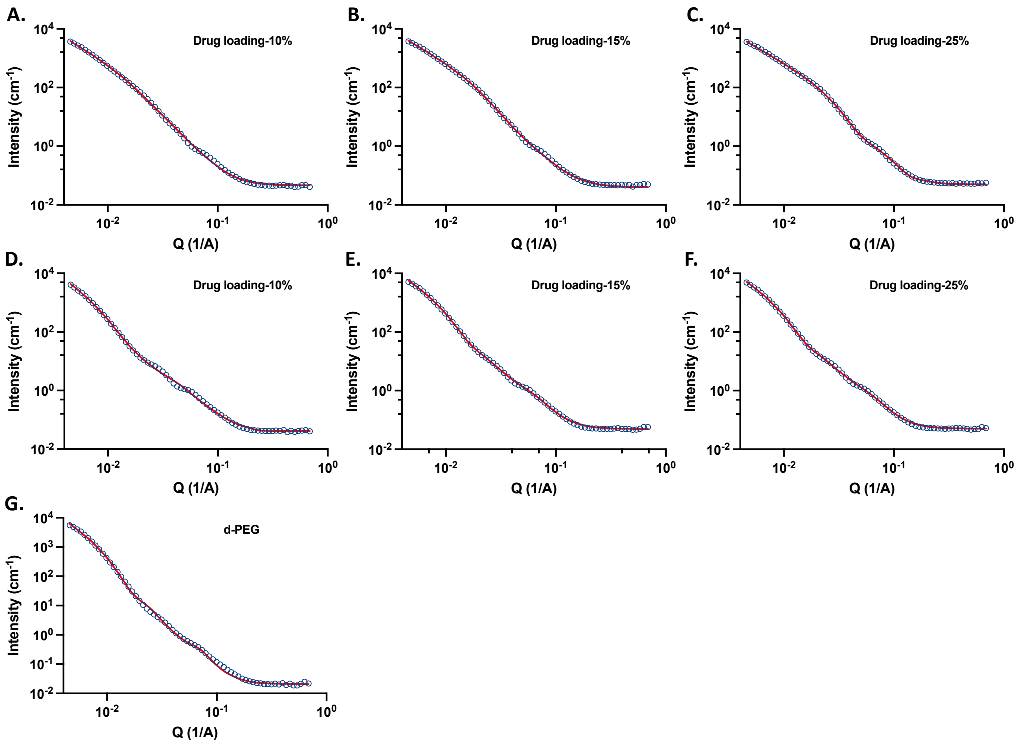

Figure S2. SANS data with fitted curves for: (A-C) PXM solid dispersions prepared by the melting method; (D-F) PXM solid dispersions prepared by the solvent evaporation method; (G) pure d-PEG.

Table S1. The potential energy of drug-drug, polymer-polymer, and drug-polymer for different drug loadings under the melting and solvent evaporation methods.

	Drug loading	Drug – Drug (KJ/mol)	Polymer – Polymer (KJ/mol)	Drug – Polymer (KJ/mol)
Melting method	10%	-18167.3	-161148	-42045.9
	15%	-32650.7	-156938	-56060
	25%	-83043.6	-146265	-87186.7
Solvent evaporation method	10%	-9066.86	-154030	-47328.3
	15%	-19222.5	-148488	-65804.2
	25%	-52262	-129617	-104679
